# A study based on four immunoassays: Hepatitis C virus antibody against different antigens may have unequal contributions to detection

**DOI:** 10.1186/s12985-021-01608-x

**Published:** 2021-07-03

**Authors:** Xinyi Jiang, Le Chang, Ying Yan, Huimin Ji, Huizhen Sun, Fei Guo, Lunan Wang

**Affiliations:** 1 National Center for Clinical Laboratories, Beijing Hospital, National Center of Gerontology, Institute of Geriatric Medicine, Chinese Academy of Medical Sciences, No.1 Dahua Road, Beijing, 100730 People’s Republic of China; 2 Beijing Engineering Research Center of Laboratory Medicine, Beijing Hospital, Beijing, People’s Republic of China; 3 Graduate School, Peking Union Medical College, Chinese Academy of Medical Sciences, Beijing, People’s Republic of China

**Keywords:** Hepatitis C virus, Anti-HCV immunoassays, HCV antigens, Blood donor screening strategy

## Abstract

**Background:**

All commercial Hepatitis C virus antibody (anti-HCV) assays use a combination of recombinant antigens to detect antibody response. Antibody responses to individual antigenic regions (core, NS3/4 and NS5) used in assays have not been investigated.

**Methods:**

In this study, we quantified HCV viral load, tested anti-HCV with four commercial assays (Ortho-ELISA, Murex-ELISA, Architect-CMIA and Elecsys-ECLIA) in 682 plasma specimens. In antigenic region ELISA platform, microwells were coated with three antigens: core (c22-3), NS3/4 (c200) and NS5 individually. The signal-to-cutoff (S/Co) values of different assays, and antibody responses to individual antigens were compared. The specimens were divided into HCV RNA positive group, anti-HCV consistent group, and anti-HCV discrepant group.

**Results:**

Anti-core and anti-NS3/4 were simultaneously detected in 99.2% of HCV RNA positive specimens and showed great consistency with total anti-HCV signals. Responses to the core region were more robust than those to the NS3/4 region in anti-HCV consistent group (*p* < 0.001). Anti-NS5 only occurred in companying with responses to the core and NS3/4 antigens, and failed to affect the final anti-HCV positive signals. In anti-HCV discrepant group, 39.0% of positive signals could not be traced back to any single antigenic region.

**Conclusion:**

Antibody responses to the core and NS3/4 antigens were stronger, whereas responses to the NS5 antigen were the weakest, indicating that individual antigenic regions played different roles in total anti-HCV signals. This study provides an impetus for optimizing commercial anti-HCV assays.

**Supplementary Information:**

The online version contains supplementary material available at 10.1186/s12985-021-01608-x.

## Introduction

Chronic hepatitis C virus (HCV) infection is a public health problem worldwide. In 2015, the World Health Organization (WHO) estimated that 1% (71 million) of the world population was infected with HCV and only 1/5 were aware of their infectious status [1]. For many people the early symptoms of HCV are mild, however, long-term follow-up have shown that the disease can further progress into complications like cirrhosis, liver failure and hepatocellular carcinoma. HCV caused 580,000 deaths in 2017 [2]. From 2000 to 2017, the mortality of HCV-attributable cirrhosis increased by 17.4% and hepatocellular carcinoma (HCC) by 30.4% [3], contributing to the increasing burden of the disease.

WHO set the goals for HCV elimination with an 80% reduction in new infections and 65% reduction in liver-related mortality by 2030 [4]. To attain these targets, 90% of chronically infected cases should be diagnosed and 80% should be treated. Individuals infected with HCV should, therefore, be identified [5]. Accurate, efficient and cost-effective screening and diagnosis of HCV infection is critical for further therapeutic decisions.

HCV infection is diagnosed using HCV RNA and HCV antibodies (anti-HCV) specific to antigens, such as structural core antigen and other non-structural proteins [6–8]. For primary screening of HCV infection, it is recommended that anti-HCV be detected. Enzyme immunoassay (EIA) is one of the widely-used methods for detecting anti-HCV. The first commercially available EIA used only recombinant peptide (c100-3) [9], which detected antibodies against the NS4 region. The seroconversion period from the incident infection to the detection of anti-HCV was 4–6 months and the false-positive rate was up to 70% in blood donors from low endemic areas [10]. To overcome these drawbacks, second-generation assays contained recombinant antigen from the NS3 (c33c) and NS4 (5-1-1p) regions together with an antigen (c22-3) from the core region, shortening the average seroconversion period to 10 weeks [11]. Third-generation anti-HCV tests included core antigens, combined NS3 and NS4 antigens and incorporated an NS5 epitope, display significantly improved performance in both sensitivity and specificity with a seroconversion period of approximately 8 weeks [12, 13]. The commercial assays currently available are predominantly based on the third-generation anti-HCV tests. However, the composition of each antigenic component and antigen size in these assays vary between manufacturers. Chemiluminescence immunoassay (CIA) is another primary screening method for anti-HCV. Performance comparisons between different CIAs with various assay formats showed great agreement for anti-HCV detection [14]. Because of its technical simplicity, full automation and greater positive predictive value (PPV), the CIA is replacing the EIA method in some high-volume clinical laboratories [15]. Regardless of the format used, the CIA or the EIA screening tests have generated notable discrepant results, particularly in low-prevalence populations, with supplemental assays for anti-HCV confirmation always required. Recombinant immunoblot assay (RIBA) 3.0 was recommended in early days by FDA as supplemental confirmatory tests due to its high specificity. However, it was discontinued and withdrawn from the market in 2010. With the availability of molecular tests, RIBA was largely obsolete in clinical practices and no longer recommended as a supplemental test by the US Centers for Disease Control and Prevention (CDC) [16]. Based on new Food and Drug Administration (FDA) guideline released in 2019 [17], HCV infection status should now be determined using an anti-HCV assay, followed by nucleic acid testing (NAT) if the anti-HCV assay is reactive. Positive results for anti-HCV and HCV RNA test indicate current HCV viremia. However, specimens with anti-HCV positive and HCV RNA negative results, suggest several possibilities, such as previously resolved HCV infection or false positivity [18]. We cannot distinguish between these outcomes because all available assays applying a combination of recombinant antigens (multi-target formats), and the antigen that contributes most to the final positive result is unknown.

Currently, there is no FDA-approved supplemental anti-HCV confirmatory assay. The guideline recommends testing first-round anti-HCV-positive specimens using another approved anti-HCV screening assay as an alternative supplemental method for anti-HCV confirmation. This FDA recommendation has been used in HCV true prevalence studies [19] and to differentiate resolved HCV infection from false anti-HCV positivity, because a false biological result should not occur with two different assays [16].

In this study, we collect serum specimens screened positive for anti-HCV, retest and compare their anti-HCV signals with four commercial assays, and further trace the positive signals based on antigenic region ELISAs. The strategy to retest anti-HCV with a second screening assay was also evaluated in all the specimens.

## Materials and methods

### Specimens collection

**Fig.1:**
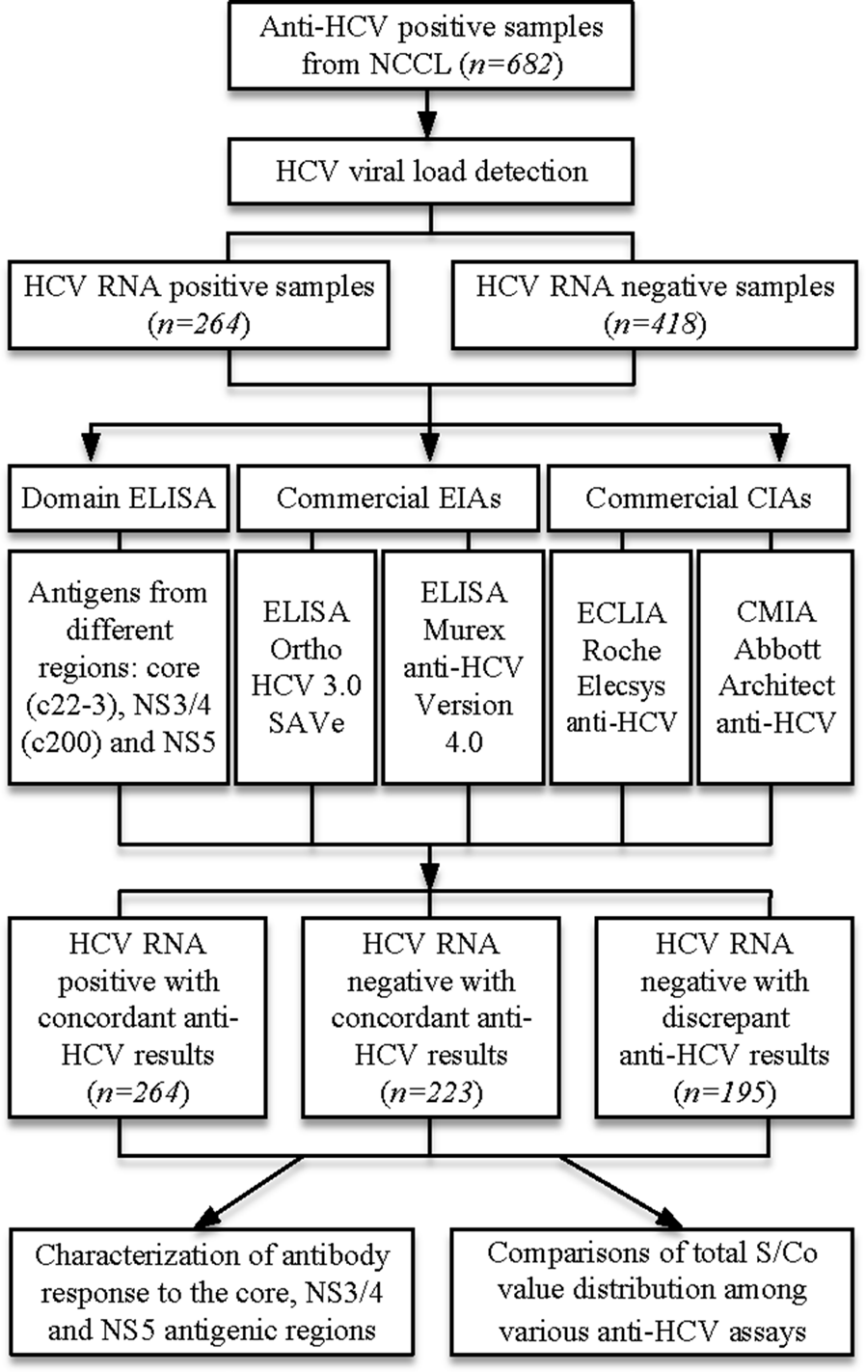
Schematic illustration of the analytical steps on anti-HCV assays. 682 anti-HCV positive samples were collected. The samples were retested anti-HCV with four assays independently. With the results of anti-HCV and HCV viral load, the samples were divided into three groups: (1) HCV RNA positive group (n = 264); (2) HCV RNA negative, anti-HCV consistent group (n = 223); (3) HCV RNA negative, anti-HCV discrepant group (n = 195). *NCCL* National Center for Clinical Laboratories, *ELISA* enzyme linked immunosorbent assay, *ECLIA* electro chemiluminescent immunoassay, *CMIA* chemiluminescent microparticle immunoassay

Between November 2012 and December 2017, 682 specimens from voluntary blood donors tested positive for anti-HCV were collected in 22 blood centers/banks from 17 provinces and municipalities including Beijing, Chongqing, Gansu, Hebei, Heilongjiang, Henan, Hubei, Hunan, Inner Mongolia, Jiangsu, Jilin, Liaoning, Ningxia, Shaanxi, Shandong, Shenzhen and Tianjin. These specimens were primary screened with EIA assays mentioned previously [20] and then transported on dry ice to National Center for Clinical Laboratories (NCCL) in Beijing. Figure 1 demonstrated the flow chart representing the study process.

### Detection of hepatitis C viral load

The Real-Time HCV assay (Abbott Diagnostics, USA) was performed to quantify the HCV viral load in all the specimens. The assay had a linear range of 12 IU/mL (1.08 log IU/mL) to 100 million IU/mL (8.0 log IU/mL). The Limit of Detection (LoD) of this assay is 12 IU/mL (1.08 log IU/mL), equivalent to the lower limit of quantitation (LLQ). The sensitivity for the assay was 12 IU/mL for the 0.5 mL specimen volume and the specificity was 99.5%. HCV viral load ≥ 1.08 log IU/mL was considered as positive.

### Anti-HCV assays

Collected specimens were independently tested with four well-recognized anti-HCV assays including two EIAs and two CIAs (Additional file 1). EIAs utilized in this study were Ortho HCV 3.0 SAVe ELISA Test System (Ortho-Clinical Diagnostics) and Murex anti-HCV Version 4.0 (DiaSorin Diagnostics). The Ortho system (Ortho-ELISA) contained three yeast produced recombinant antigens (Grifols Corporation, CA, USA) c22-3, c200 and NS5, representing the core, NS3/4 and NS5 regions of the HCV genome respectively. In Murex anti-HCV assay (Murex-ELISA), microplate were coated with highly purified antigens representing core, NS3, NS4 and NS5.

According to Ortho ELISA system, three antigens: core (c22-3), NS3/4 (c200) and NS5 were individually coated to microwells, making it possible to measure anti-HCV positive specimen reactivity to three antigens individually, which can be regarded as antigenic region ELISA. The antigenic region ELISA is a three-stage test. During incubation in the first stage, antibody binds to individual antigen and forms antigen–antibody complex on the microwell surface. In the second stage, murine monoclonal antibody conjugated to horseradish peroxidase is added and binds to the human IgG portion of the immune complexes. Enzyme detection system is added in the third stage to generate colored end products. The color intensity is measured with a microwell reader and Signal-to-Cutoff (S/Co) values are calculated to measure the concentration of specific antibody. Antigenic region ELISA, Ortho and Murex EIAs’ results can all be expressed as S/Co values. The specimens with a result of S/Co values ≥ 1.0 was considered as reactive in EIAs as well as in CIAs.

CIAs used in this study are Architect anti-HCV assay (Abbott Laboratories) and Elecsys anti-HCV assay (Roche Molecular Diagnostics). The anti-HCV tests were performed using the Architect i2000 and Cobas e 411 analyzer respectively. In Architect system (Architect-CMIA), the E.coli produced HCr43 was a fusion protein of HCV genomic coding region core and NS3. It also contained a yeast produced NS4 protein of c100-3 in Architect anti-HCV assay. Specimens with S/Co ≤ 0.79 were nonreactive and there was no need for retesting. Specimens in gray zone (S/Co 0.8–0.99) should be retested in duplicates. If no reactivity was found in both cases, the specimen was anti‑HCV negative. The Elecsys Anti‑HCV II assay (Elecsys-ECLIA) was a third-generation test and used peptides and recombinant proteins representing HCV core, NS3 and NS4 antigens to determine anti‑HCV. Specimens with S/Co < 0.9 are nonreactive in the Elecsys-ECLIA while specimens with S/Co ≥ 0.9 and < 1.0 were considered borderline and should be redetermined. When no reactivity was found in both duplicates, the specimen could be reported as negative.

### Statistical analysis

The statistical analysis and representation were performed using SPSS 21.0 and GraphPad Prism 7.0 software. The mean S/Co values in four anti-HCV assays among groups with different HCV serological patterns were compared with one-way ANOVA followed by Fisher's least significant difference test. The distribution of S/Co value in antigenic region ELISAs were compared with one-way ANOVA followed by Tukey's multiple comparisons test. The sensitivity and specificity were calculated with 95% confidence interval. The statistical significance was defined as *p* < 0.05.

## Results

### Comparing the four anti-HCV assays

The four assays showed great performance in diagnosing viremia specimens (Table 1) and all 264 HCV RNA(+) specimens displayed positive anti-HCV results. In this HCV RNA(+) group, the two CIA assays generated overall higher anti-HCV S/Co values than the two EIA assays. The S/Co values in this group were all greater than 4 using Elecsys-ECLIA. Except one sample between 1 and 2.5, the S/Co values were all greater than 4 using Ortho-ELISA. Except two samples between 2.5 and 4, the S/Co values were all greater than 4 using Architect-CMIA. The S/Co values from Murex-ELISA were higher than 4 in 213 specimens (80.7%), between 2.5 and 4 in 29 specimens (11.0%), and between 1 and 2.5 in 22 specimens (8.3%).

**Table 1:** HCV infection status in relation to four S/Co ranges in four assays

Assays	Groups	S/Co ranges			
		< 1	1–2.5	2.5–4	> 4
Ortho ELISA	Viremia (n)	0	1	0	263
	%	0.0	0.4	0.0	99.6
	Consistent (n)	0	47	34	142
	%	0.0	21.1	15.2	63.7
	Discrepant (n)	80	73	26	16
	%	41.0	37.4	13.3	8.2
Murex ELISA	Viremia (n)	0	22	29	213
	%	0.0	8.3	11.0	80.7
	Consistent (n)	0	29	21	173
	%	0.0	13.0	9.4	77.6
	Discrepant (n)	86	41	17	51
	%	44.1	21.0	8.7	26.2
Architect CMIA	Viremia (n)	0	0	2	262
	%	0.0	0.0	0.8	99.2
	Consistent (n)	0	70	20	133
	%	0.0	31.4	9.0	59.6
	Discrepant (n)	29	92	47	27
	%	14.9	47.2	24.1	13.8
Elecsys ECLIA	Viremia (n)	0	0	0	264
	%	0.0	0.0	0.0	100.0
	Consistent (n)	0	0	0	223
	%	0.0	0.0	0.0	100.0
	Discrepant (n)	152	2	3	38
	%	77.9	1.0	1.5	19.5

^a^In each of the four assays, specimens were classified into three groups: (1) HCV RNA positive group (Viremia, n = 264); (2) HCV RNA negative, anti-HCV consistent group (Consistent, n = 223); (3) HCV RNA negative, anti-HCV discrepant group (Discrepant, n = 195). In each group, the specimens were further divided into four S/Co ranges with specific numbers and percentages. S/Co value < 1.0 was considered as anti-HCV negative while S/Co value > 4 was considered strongly anti-HCV positive

**Fig.2:**
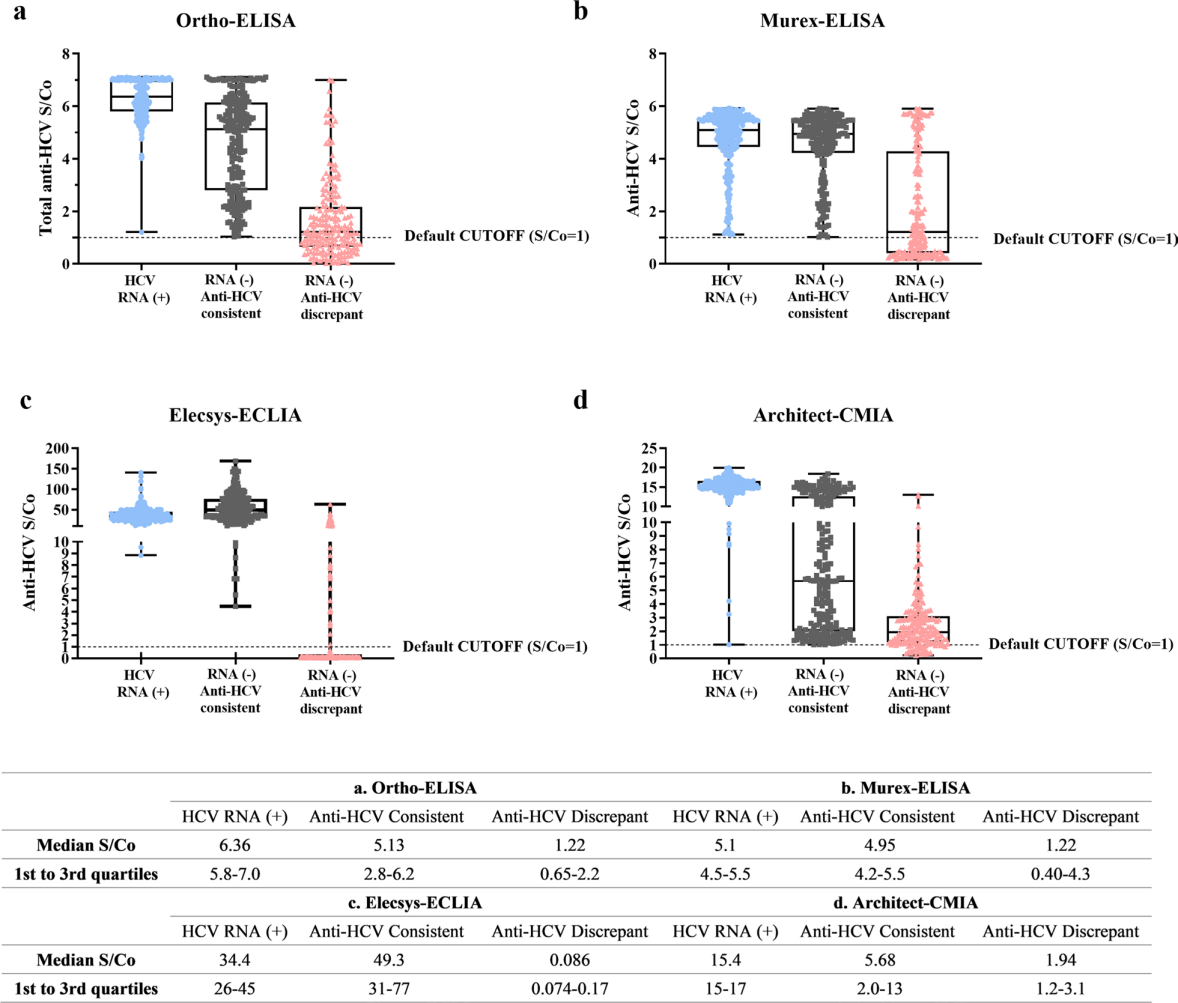
S/Co value distribution in four anti-HCV assays. Distribution of the Signal-to-Cutoff (S/Co) values in Ortho-ELISA (**a**), Murex-ELISA (**b**), Elecsys-ECLIA (**c**) and Architect-CMIA (**d**) assays were shown. The default cutoff value is 1.0 and S/Co ≥ 1 is regarded as positive. S/Co values were higher in chemiluminescent assays (Elecsys-ECLIA and Architect-CMIA) than in enzyme immunoassays (Ortho-ELISA and Murex-ELISA) due to colorimeter high-end reading limitations

For the serologically consistent group with HCV RNA(−) but that was determined to be anti-HCV(+) by all four assays (n = 223), donors were most likely infected in the past and carrying true anti-HCV antibodies. S/Co distribution among the four assays shifted to a lower value range than the HCV RNA(+) group’s (Fig. 2). The medium S/Co between Ortho-ELISA and Murex-ELISA was equivalent (5.13 vs. 4.85, *p* = 0.5013), although Murex-ELISA distributed slightly more specimens to the higher S/Co value range. For the two CIA assays, Elecsys-ECLIA had a much higher medium S/Co value of 49.3 than Architect-CMIA, with a medium S/Co value of 5.68 (*p* < 0.001).

For the serologically discrepant group that were HCV RNA(−) and anti-HCV discrepant among the four assays (n = 195), donors’ HCV infection status was uncertain, either patient had recovered from a prolonged HCV infection in the past with residual anti-HCV being detected, or donors had no HCV infection in the first place, meaning the anti-HCV positive results were false. The S/Co distribution among four assays shifted significantly to the low-value range compared to HCV RNA(+) group and anti-HCV consistent group. Both EIAs detected many negative specimens in the anti-HCV-discrepant group, with 41.0% (Ortho-ELISA) and 44.1% (Murex-ELISA) portions. Meanwhile, Murex-ELISA identified more robust positive specimens. 26.2% (51/195) of the specimens had S/Co values greater than 4, compared to 8.2% (16/195) of Ortho-ELISA specimens (Table 1). For the two CIA assays, Architect-CMIA had a medium S/Co value of 1.94, which was higher than Elecsys-ECLIA’s 0.09 0 (*p* = 0.044) (Fig. 2). Architect-CMIA had the highest number of positives compared to the other three assays, detecting 166 positives (85.1%) versus 115/195 (59.0%) by Ortho-ELISA, 109/195 (55.9%) by Murex-ELISA and 43/195 (22.1%) by Elecsys-ECLIA. Architect-CMIA also detected 27 strong positives (13.8%) with S/Co values greater than 4, which falls between the percentages for strong positives in Ortho-ELISA (8.2%) and Murex-ELISA (26.2%).

Elecsys-ECLIA detected the fewest number of positive specimens (n = 43) in this discrepant group, while other three assays all determined more than 55% of the 195 specimens as positive. When the four assays were performed as secondary assays to evaluate the positives determined by other assays, the positives by Ortho-ELISA, Murex-ELISA and Architect-CMIA received low agreement to the Elecsys-ECLIA with 13.0%, 28.4% and 10.2% respectively. On the other hand, Ortho-ELISA, Murex-ELISA and Architect-CMIA achieved positive agreement above 50% with each other in Table 2.

**Table 2:** Positive agreement rate between two assays in anti-HCV discrepant group

1st assay	Positive results in 195 specimens		Agreed by 2nd assay of							
	n	%	Ortho ELISA		Murex ELISA		Architect CMIA		Elecsys ECLIA	
			n	%	n	%	n	%	n	%
Ortho-ELISA	115	59.0			61	53.0	102	88.7	15	13.0
Murex-ELISA	109	55.9	61	56.0			88	80.7	31	28.4
Architect -CMIA	166	85.1	102	61.1	88	52.7			17	10.2
Elecsys-ECLIA	43	22.1	15	34.9	31	72.1	17	39.5		

### Antibody response pattern in antigenic region ELISAs

Three individual antigens, representing the HCV antigenic region of the core, NS3/4, and NS5, were coated to develop HCV antigenic region ELISA, in order to trace assay signals from positive specimens in three serologically characterized groups.

For the HCV RNA(+) group (n = 264), majority of the specimens (99.2%, 262/264) demonstrated reactivity to at least two HCV antigenic regions. Among them, 134 (51.1%) reacted to all three antigenic regions and 128 (48.9%) were reactive to core and NS3/4 region. One specimen reacted only to NS3/4 antigen with an S/Co value of 5.79. No specimen reacted only to the core or the NS5 antigen. Remarkably, one specimen had an S/Co value below 1 in response to all single antigens (Additional file 2). An elevated signal was observed in response to the core antigen (S/Co = 0.78), a low signal to the NS3/4 antigen (S/Co = 0.21) and no signal to the NS5 antigen (S/Co = 0.04). To this “all-single-antigen-negative” specimen, the combined reactivity to three antigenic regions eventually contributed to anti-HCV positivity in all four assays (S/CO = 1.21 by Ortho-ELISA, S/Co = 1.12 by Murex-ELISA, S/Co = 1.02 by Architect-CMIA and S/Co = 9.53 by Elecsys-ECLIA).

**Fig.3:**
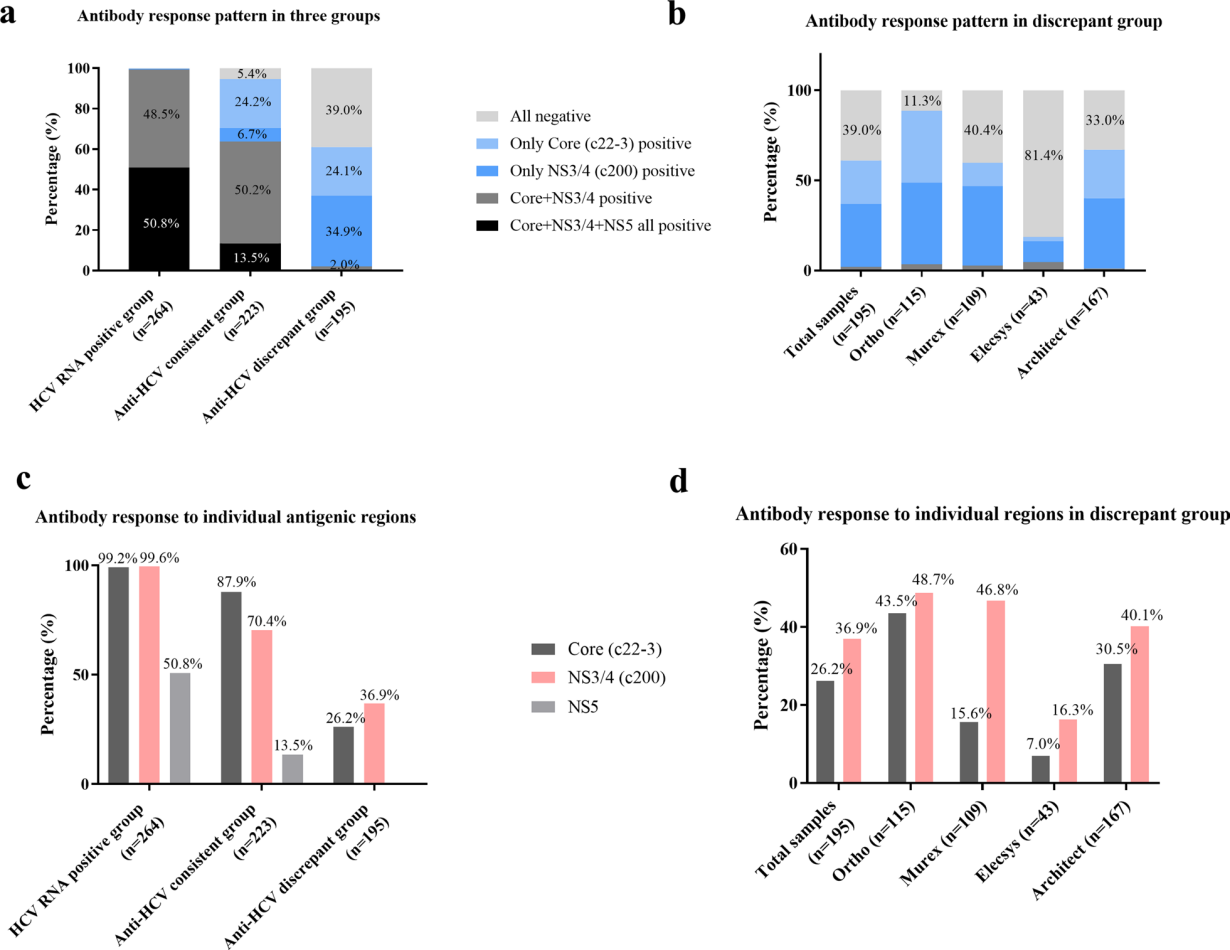
Individual and combination of antibody response to different regions. Five antibody response patterns, indicated by percentage, were compiled in column format for each of the three HCV serological groups (**a**); the compiled antibody response patterns in serological discrepant group were displayed for each of the four assays (**b**); Antibody response to individual core, NS3/4 and NS5 antigenic regions were showed in percentage in three serological groups (**c**), as well as in the serological discrepant group by each of the four assays (**d**)

For the anti-HCV consistent group (n = 223), 30 specimens showed reactivity to all three antigenic regions and 112 specimens reacted to both the core and NS3/4, with a combined reactivity of 63.7% (13.5% + 50.2%), significantly lower than 99.2% observed in HCV RNA(+) group with the same reactivity pattern (*p* < 0.001) (Fig. 3a). Fifty-four (24.2%) specimens only reacted to the core antigen and 15 (6.7%) only reacted to the NS3/4 antigen. No specimen reacted only to the NS5 antigen in this group. Twelve specimens (5.4%) had an S/Co value below 1 in response to any antigens, compared to only one specimen in the HCV RNA(+) group.

For the anti-HCV discrepant group (n = 195), patients either had residual anti-HCV from past HCV infections or their anti-HCV results were false. There was no specimen in this group that showed reactivity to all three antigens, only 4 specimens (2%) showed S/Co > 1 reactivity to both core and NS3/4 antigens. Single NS3/4 antigen reactive specimens increased to 68 (34.9%) compared to 47 (24.1%) single core antigen reactive specimens. Moreover, the uncertainty of patients’ anti-HCV status became apparent because 76 (39.0%) specimens had S/Co values below 1 in reaction to all three antigens (Fig. 3).

**Fig.4:**
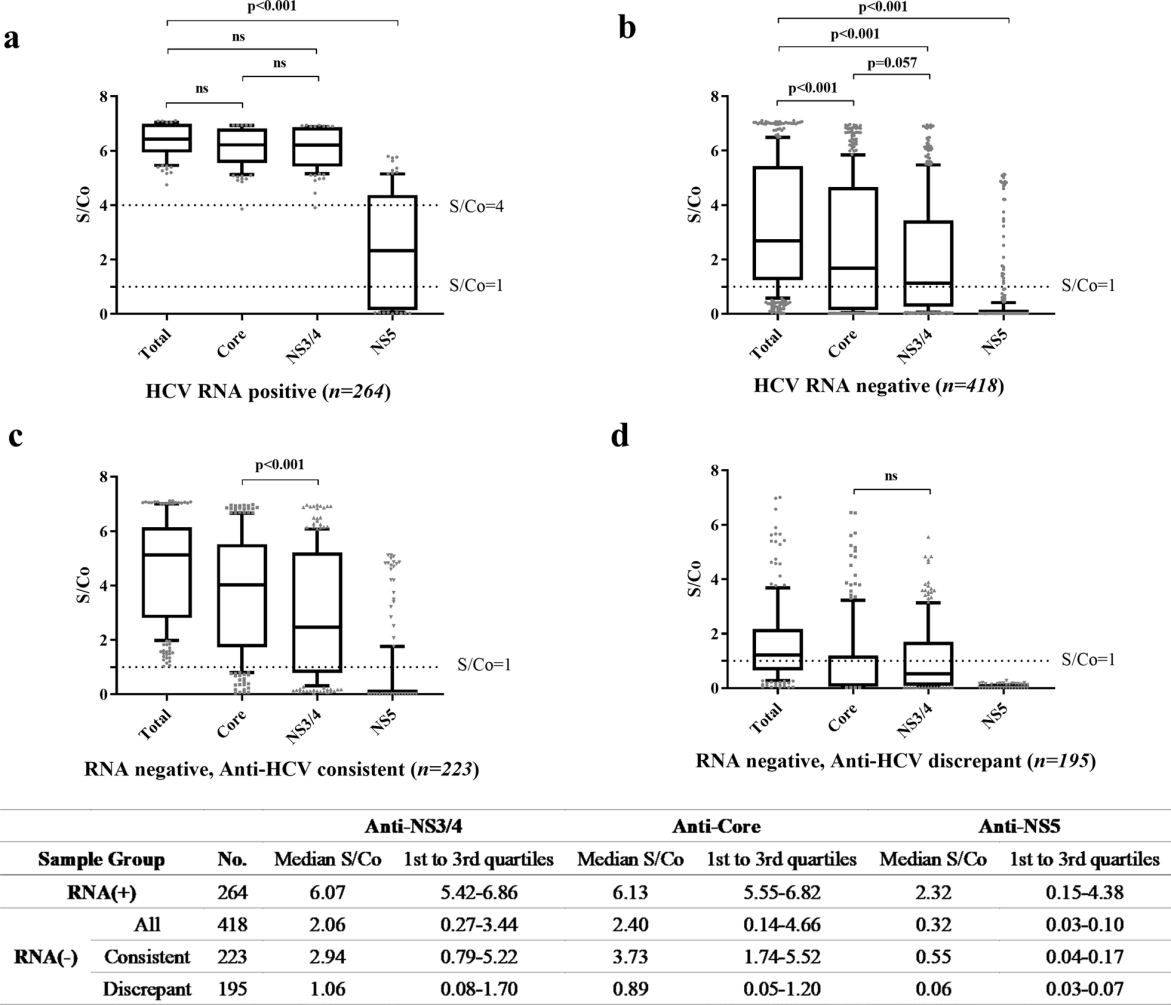
Analysis of antibody response to core, NS3/4 and NS5 antigenic regions. Antibody response to the HCV core, NS3/4 and NS5 antigenic regions were analyzed in HCV RNA positive group (**a**), HCV RNA negative group (**b**), HCV RNA negative, anti-HCV consistent group (**c**) and HCV RNA negative, anti-HCV discrepant group (**d**). The default cutoff value is 1.0 and S/Co ≥ 1 is considered as positive. *ns* not significant

Several notable observations were made to scrutinize all 682 assay reactive responses to each antigenic region: (1) Responses to the NS5 region were minimum, only occurring alongside responses to the core and NS3/4 antigenic regions and only in the HCV RNA(+) group and anti-HCV consistent group. There was no response to the NS5 alone nor in the company of another single antigen in any of the three groups; (2) Responses to core and NS3/4 were comparable and showed great consistency with total anti-HCV signals in viremia specimens (Fig. 4). Specimens showed higher response to core region than to NS3/4 region in anti-HCV consistent group (*p* < 0.001), while in the anti-HCV discrepant group, response signals to NS3/4 region were equal to core region (*p* = 0.5017); (3) The original positive signal (as high as 39.0% in the anti-HCV-discrepant group) of many RNA negative specimens generated by product assay could not be traced back to any single antigenic region. This percentage decreased to 18.5% (36/195) when S/Co values were lowered by 50% from > 1 as positive to > 0.5 as positive in the antigenic region ELISA.

## Discussion

The prototype of Ortho 3.0 ELISA system was released in 1993 [21]. In this system, the recombinant core protein was named c22-3, the recombinant NS3 (c33c) and NS4 (c100-3) proteins were fused into a new protein c200. c22-3, c200 and NS5 recombinant antigens were coated on microplates to form the anti-HCV detection platform [22].

In vivo, core antigens triggered earliest antibody response more frequently [23]. Anti-core generated shortly after seroconversion and reacted to the coated c22-3 antigens. c200, covering the entire NS3 helicase region and most of the NS4 region, improved the sensitivity of third-generation HCV screening methods and detected a strong signal of anti-NS3/4 early in the serological conversion. It also persisted during resolved HCV infection [24], however, the responses were higher in individuals with viral persistence than those with viral clearance [25]. Our results supported the notion that anti-core and anti-NS3/4 are two significant indicators of HCV infection. 99.2% of the viremia patients had both anti-core and anti-NS3. On the other hand, anti-NS5 accounted for 50.8% in viremia specimens, and all the anti-NS5 reactive specimens had anti-core and anti-NS3 seroconverted.

The NS5 antigen was poorly immunogenic, and responses to NS5 were generally the lowest and likely to be missing [25, 26]. Incorporation of NS5 antigen contributed much less to the overall assay sensitivity [27]. Although anti-NS5 was detected in a proportion of patients with HCV infections [28], two ELISAs that incorporated NS5 antigen demonstrated no sensitivity advantage over two CIAs that did not contain NS5 antigen in this research. Anti-NS5 failed to affect the final anti-HCV positive signals compared to core and NS3/4 antibodies; anti-NS5 was also not the reason for anti-HCV discrepancy, given that anti-NS5 disappeared in the serologically discrepant group.

In this study, we divided the specimens into three groups according to their serological indicators. We found different antibody response patterns related to the production and degradation of individual antibodies. Two patterns (all three antigen positive and core + NS3/4 positive) were observed in HCV RNA(+) group with comparable proportions (Fig. 3a). The simultaneous positivity of three antibodies may be an indication of current and early infection. Individual core and NS3/4 antigenic region dominated the anti-HCV response in HCV RNA(+) group and anti-HCV consistent group (Fig. 3c). But all positive patterns decreased in the anti-HCV consistent specimens and the only detection of anti-core increased, reflecting the natural proceeding of the infection, either core antigen is more resistant to protein degradation or anti-core has a slow decay rate upon resolving infection. From anti-HCV consistent group to discrepant group (Fig. 3), antigen stimulation disappeared but the only anti-NS3/4 reactive pattern increased from 6.7 to 34.9%. Positive signals of anti-NS3/4 declined with a smaller proportion than anti-core’s. In this discrepant group, anti-NS3/4 showed a more considerable portion share than anti-core among all four assays. The mechanism underlying the persistence and decay of HCV antibodies remains unknown. However, two models on long-term antibody responses may explain our findings. One model is the persisting antigen model [29], in which HCV core antigens may exist on the surface of dendritic cells in the form of immune complexes, and these complexes can constantly stimulate memory B cells, generate plasma cells, and then secrete anti-core antibodies. Another is the “imprinted lifespan” model [29, 30], where the persistence of an antibody would be associated with the lifespan of antigen-specific plasma cells. NS3/4 antigens may induce B cell stimulation more potently and differentiated plasma cells may be imprinted with longer lifespans. As a result, anti-NS3/4 can be produced continuously and exist in the patients for a longer period.

In HCV RNA(+) group, one specimen revealed an obvious NS3 seroconversion and core immune response was not detected, which may be due to a delayed response (Additional file 2). Another viremia specimen had miniscule immune response to core and NS3 by antigenic region ELISA, but was barely detected positively by all four assays. The S/Co value of 9.5 by Elecsys-ECLIA was significantly higher than the low S/Co values (between 1.02 and 1.21) by the other three assays. These immense S/Co value differences could be attributed either to a better NS3 antigen used in Elecsys, or higher specimen volume used in this assay format. Elecsys-ECLIA adopted an antigen sandwich format while the other three assays employed an indirect assay format. Antigen sandwich format delivers higher sensitivity because it allows larger specimen volume in reaction, while in indirect format, specimen in reaction is usually below 10% of the total volume to avoid excessive human IgG that could cause high background by anti-human IgG conjugates.

The four anti-HCV assays in our study were appropriate for routine use in the reliable detection of anti-HCV. Employing a secondary anti-HCV assay different from the first one can be reliable in predicting resolved HCV infection. Reactive specimens, as tested by two assays, did not need to undergo supplementary anti-HCV confirmation. But confirming the infectious status in anti-HCV discrepant group was challenging because the antibody responses were complex and untraceable. False positive anti-HCV results most commonly occurred in populations with a low prevalence of HCV infection [16]. Considering the epidemiology of HCV in the region, caution must be exercised in deciding the sequence of the assays. Architect-CMIA showed the highest positive rates in discrepant specimens and achieved good agreement rates when applied as the second assay (Table 2). To elevate the agreement rates, we recommended to apply assays with higher positive rates as the secondary tested one. The strategy of using two anti-HCV assays with good agreement may increase predictions of resolved infections. By employing assays with higher positive rates as the secondary ones, higher agreement rates can be achieved to confirm the first ones. However, for the specimens with inconsistent anti-HCV results, confirmation experiments are still required to determine true or false positivity.

Despite its promising findings, this study had some limitations. Firstly, we conducted a cross-sectional investigation and did not follow up the patients. Because monitoring the process of natural infection, production and decay of antibody was challenging, and available reports on the persistence and decline of anti-HCV were few. However, we believe that a longitudinal study is of great importance and must be conducted for further research. Secondly, the NS5 antigen failed to affect final anti-HCV positive signals compared to core and NS3/4, the escape from detection, and the preparation of the NS5 antigen could be responsible and need further investigation. Thirdly, a large number of RNA negative specimens (39.0%) in the anti-HCV discrepant group could not be traced back to any single antigenic region. These positive signals’ origins remained unclear. Subdividing antigens into smaller antigenic epitopes and conducting more specific antigenic region ELISA could help differentiate changes in the individual protein region during the course of HCV, potentially providing directions for further study.

## Conclusion

We found that using two anti-HCV assays with good agreement could increase the chances of predicting resolved infections. Moreover, antibody responses to individual antigenic regions could play different roles in total anti-HCV signals and showed different patterns during the course of HCV. We further revealed the relations between total signals and individual antigenic regions (core, NS3/4 and NS5), which may help us gain insight into the natural history of HCV. Core and NS3/4 are strong immunogenic proteins while responses to NS5 are the weakest, which may help manufacturers adjust antigen proportions to optimize quality of commercial assays.

## Supplementary Information

**Additional file 1**. Information and positive rates of four anti-HCV assays.

**Additional file 2**. Numbers for reactive response to individual core, NS3/4 and NS5 antigenic regions in three serological groups.

## Data Availability

Data sharing is not applicable to this article as no datasets were generated or analyzed during the current study.

## Abbreviations

HCV: Hepatitis C virus
Anti-HCV: Hepatitis C virus antibody
EIA: Enzyme immunoassay
ELISA: Enzyme linked immunosorbent assay
CIA: Chemiluminescence immunoassay
ECLIA: Electro chemiluminescent immunoassay
CMIA: Chemiluminescent microparticle immunoassay
WHO: World Health Organization
PPV: Positive predictive value
RIBA: Recombinant immunoblot assay
CDC: Centers for Disease Control and Prevention
FDA: Food and Drug Administration
NAT: Nucleic acid testing
S/Co: Signal-to-cutoff
HCC: Hepatocellular carcinoma
