# Supplementary material for: A study based on four immunoassays: Hepatitis C virus antibody against different antigens may have unequal contributions to detection
Source: Virol J. 2021 Jul 3;18:137. doi: 10.1186/s12985-021-01608-x (PMC8255013; doi:10.1186/s12985-021-01608-x)
Supplement: Supplementary file 1 — Additional file 1. Information and positive rates of four anti-HCV assays. [file 12985_2021_1608_MOESM1_ESM.docx]

**Additional file 1**. **Information and positive rates of four anti-HCV assays**

| **Assays** | **Antigen coated** | **Analyzer** | **Manufacturer** | **Assay principle** | **Anti-HCV positive samples** | **Anti-HCV positive rate** |
| --- | --- | --- | --- | --- | --- | --- |
| Ortho HCV Version 3.0 ELISA Test System | putative core (c22-3), NS3/4 (c200)  and NS5 | Manual | Ortho-Clinical Diagnostics | Enzyme linked immunosorbent assay (ELISA) | *602* | *88.3%* |
|  | Core antigen |  |  |  | *509* | *74.6%* |
|  | NS3/4 antigen |  |  |  | *492* | *72.1%* |
|  | NS5 antigen |  |  |  | *164* | *24.0%* |
| Murex anti-HCV Version 4.0 | Core, NS3, NS4 and NS5 | Manual | DiaSorin Diagnostics | Enzyme linked immunosorbent assay (ELISA) | *596* | *87.4%* |
| Elecsys Anti-HCV Ⅱ | Core, NS3 and NS4 | Cobas e 411 | Roche Diagnostics | Electro chemiluminescent immunoassay (ECLIA) | *518* | *76.0%* |
| Architect Anti-HCV | HCr43 fusion protein (core and NS3)  and NS4 (c100-3) | Architect i2000 | Abbott Laboratories | Chemiluminescent microparticle immunoassay (CMIA) | *654* | *95.9%* |

In this study, all the specimens were tested by four well-recognized anti-HCV immunoassays. Information (the coated antigen, the way of analyzer, the principle and manufacturer) and positive rates of each immunoassay was provided above.
