# Supplementary material for: A study based on four immunoassays: Hepatitis C virus antibody against different antigens may have unequal contributions to detection
Source: Virol J. 2021 Jul 3;18:137. doi: 10.1186/s12985-021-01608-x (PMC8255013; doi:10.1186/s12985-021-01608-x)
Supplement: Supplementary file 2 — Additional file 2. Numbers for reactive response to individual core, NS3/4 and NS5 antigenic regions in three serological groups. [file 12985_2021_1608_MOESM2_ESM.docx]

**Additional file 2**. **Numbers for reactive response to i****ndividual core, NS3/4 and NS5 antigenic regions in three serological groups**

|  | Core+NS3/4+NS5 | Core+NS3/4 | NS3/4 only | Core only | NS5 only | All negative | Total |
| --- | --- | --- | --- | --- | --- | --- | --- |
| HCV RNA positive group (Number) | **134** | **128** | **1 (Core: 0.03; NS3/4: 5.79; NS5: 0.03 )** | **0** | **0** | **1  (Core: 0.78; NS3/4: 0.21; NS5: 0.04)** | **264** |
| Ortho (S/Co) |  |  | 7.04 |  |  | 1.21 |  |
| Murex (S/Co) |  |  | 5.58 |  |  | 1.12 |  |
| Elecsys (S/Co) |  |  | 50.64 |  |  | 9.53 |  |
| Architect (S/Co) |  |  | 9.5 |  |  | 1.02 |  |
| Anti-HCV consistent group (Number) | **30** | **112** | **15** | **54** | **0** | **12** | **223** |
| Anti-HCV discrepant group (Number) | **0** | **4** | **68** | **47** | **0** | **76** | **195** |
| Ortho (Number) | 0 | 4 | 52 | 46 | 0 | 13 | 115 |
| Murex (Number) | 0 | 3 | 48 | 14 | 0 | 44 | 109 |
| Elecsys (Number) | 0 | 2 | 5 | **1** | 0 | 35 | 43 |
| Architect (Number) | 0 | 2 | 65 | 45 | 0 | 55 | 167 |

This table shows different response pattens to three individual antigenic regions. In HCV RNA positive group, 134 specimens were reactive to core, NS3/4 and NS5 antigens, 128 specimens were reactive to core and NS3/4 antigens. One specimen was only reactive to NS3/4 antigen. This specimen was tested positive by four immunoassays, the S/Co value was 7.04 by Ortho-ELISA, 5.58 by Murex-ELISA, 50.64 by Elecsys-ECLIA, 9.5 by Architect-CMIA. Only one specimen had an S/Co value below 1 in response to all single antigens. This specimen was also tested positive by four immunoassays, the S/Co value was 1.21 by Ortho-ELISA, 1.12 by Murex-ELISA, 9.53 by Elecsys-ECLIA, 1.02 by Architect -CMIA. In anti-HCV discrepant group, not only the numbers for different response pattens are shown, but the reactive numbers for each immunoassay are also listed.
